# Plastic responses to past environments shape adaptation to novel selection pressures

**DOI:** 10.1073/pnas.2409541122

**Published:** 2025-01-30

**Authors:** Sarah E. R. Coates, Aaron A. Comeault, Daniel P. Wood, Michael F. Fay, Simon Creer, Owen G. Osborne, Luke T. Dunning, Alexander S. T. Papadopulos

**Affiliations:** ^a^Molecular Ecology and Evolution Group, School of Environmental and Natural Sciences, Bangor University, Bangor LL57 2UW, United Kingdom; ^b^Royal Botanic Gardens Kew, Richmond TW9 3AE, United Kingdom; ^c^School of Plant Biology, University of Western Australia, Crawley, WA 6009, Australia; ^d^Ecology and Evolutionary Biology, School of Biosciences, University of Sheffield, Sheffield S10 2TN, United Kingdom

**Keywords:** adaptive potential, preadaptation, gene expression, exaptation

## Abstract

Phenotypic plasticity may pave the way for rapid adaptation to newly encountered environments. Although it is often contested, there is growing evidence that initial plastic responses of ancestral populations to new environmental cues may promote subsequent adaptation. However, we do not know whether plasticity to cues present in the ancestral habitat (past-cue plasticity) can facilitate adaptation to novel cues. Conceivably, this could occur if plastic responses are coincidentally optimal to both past and novel cues (i.e., are preadaptive) or if they are transferred to novel cues during adaptation. Past plastic phenotype values could also become fixed during adaptation to the new environment. To uncover the role of past-cue plasticity in adaptation, we tested gene expression plasticity responses of two parallel mine-waste-adapted *Silene uniflora* populations and their closest coastal relatives. Plants were exposed to the past and novel cues of salt and zinc, which revealed that during adaptation to mine waste, plasticity to salt diminishes. Despite this, our results show that ancestral plasticity to salt has a substantial impact on subsequent adaptation to zinc. For a third of genes that have evolved zinc plasticity in mine populations, salt plasticity has been transferred to the zinc response. Furthermore, a quarter of fixed expression differences between mine and coastal populations were similar to ancestral salt responses. Alongside evidence that ancestral plasticity to novel cues can facilitate adaptation, our results provide a clear indication that ancestral past-cue plasticity can also play a key role in rapid, parallel adaptation to novel habitats.

## Significance

The role of phenotypic plasticity in promoting adaptation is hotly debated, with conflicting evidence for the benefits of ancestral plasticity in newly encountered environments. Here, we present an alternative mode by which ancestral plasticity can promote adaptation. We investigated whether phenotypic plasticity toward environmental cues that are experienced only in ancestral habitats (past-cue plasticity) can significantly contribute toward rapid adaptation to completely distinct cues. We show that in the maritime plant species, *Silene uniflora,* past-cue plasticity to salt has made a substantial contribution to rapid, parallel adaptation to heavy-metal pollution in newly encountered habitats. This phenomenon has broad implications for the capacity and predictability of species to persist and adapt in the face of anthropogenic environmental change.

Phenotypic plasticity is the ability of an individual genotype to produce different phenotypes in response to different environmental cues (1, 2). Although plasticity can be adaptive (i.e., it increases fitness), the extent to which it can facilitate or even lead adaptation to novel habitats remains contested (3–11). One possible outcome is that when plasticity moves a phenotype value closer to the optimum for the novel habitat, selection may act on quantitative genetic variation to change the extent of plasticity (i.e., genetic accommodation) (3, 5–7, 12, 13). Selection might promote increased environmental sensitivity (increased plasticity) or the initially plastic phenotype may become canalized and no longer vary with environment (i.e., genetic assimilation) (6, 14). Alternatively, initial plastic responses may be neutral (i.e., not under selection) or maladaptive (i.e., reduce fitness) and are reversed/reduced during adaptation (4, 15). Studies typically investigate these phenomena by focusing on whether the plastic change in an ancestral population in response to a novel environment (referred to as “PC”) moves trait values in the same direction as the evolutionary change (“EC”) in the derived population that follows adaptation (8, 11, 15, 16).

Less attention has been devoted to the impact of ancestral plastic responses to past cues (i.e., those only experienced in the ancestral environment—here termed past-cue plasticity) on subsequent adaptation to different cues in the new environment (12, 17, 18). It is often thought that existing traits with one function may serve a new, beneficial purpose in new environments (19, 20); i.e., the traits may be preadaptive. Existing phenomena suggest that tolerance to one stress may be preadaptive for additional stressors in plants—for example, cotolerance has been observed between different heavy metals (21, 22), and salt and heavy metal tolerance mechanisms may be shared (23–25). Despite the existence of cotolerance of multiple stressors, there is little direct evidence to show that past-cue plasticity can be preadaptative for novel cues.

Genetic accommodation, genetic assimilation, and preadaptive plasticity (as described above) require plastic phenotypes to be expressed on exposure to cues in the new environment in order to be visible to selection (13, 14). Here, we investigate two modes by which past-cue plasticity might facilitate adaptation without the ancestral plastic response to the new cue increasing fitness, termed “cue transfer” and “genetic adoption.”

We define cue transfer as when a plastic response to a past cue is transferred to a new cue following adaptation, a possibility supported by theoretical modeling (18). This may occur if: i) Mutations in receptors that detect ligands generated by the past cue enable them to bind ligands generated by the new cue (26), ii) mutation in cis and/or transregulatory elements alter the timing and/or tissue in which a gene is expressed/suppressed as a response to the new cue rather than past cue (27, 28). iii) On colonization, trait expression is altered by epigenetic modification (e.g., hypermethylation) allowing past-cue plasticity to be expressed in response to the new cue (29). Subsequent selection on genetic variation might then drive adaptation and adaptive refinement of the transferred trait.

We define genetic adoption as when the response trait value for the past cue in the ancestral population becomes constitutively expressed in a derived population as an adaptation to a new stressor. In other words, natural selection driven by the new stressor produces a constitutive change in the phenotype that resembles the past-cue plastic value. This might occur i) when an initial cue transfer step is followed by genetic assimilation. After the cue is transferred, adaptive refinement is also possible prior to genetic assimilation; ii) If there is indirect selection for the past-cue response in the new environment due to genetic correlations (12, 30, 31). In this case, selection in the new environment acts on a quantitative trait with underlying genetic variation. Due to genetic correlations or physiological constraints, adaptation of this trait to the new environment stimulates a change in a second, ancestrally plastic trait that is not directly responsive to cues in the new environment. For example, adaptive changes in plant height in *Phlox drummondii* can be accompanied by shifts in root or flower traits (31). If plastic values of the second trait are adaptive in the new environment, the newly exposed variation will allow the second trait to evolve and become constitutively expressed; iii) as genes underlying past-cue plasticity (even maladaptive plasticity) are likely to harbor greater levels of polymorphism due to relaxed purifying selection or because they are fast evolving (32). In this case, there may be no ancestral plastic response to the new cue, or it may be neutral or maladaptive. Following colonization of the new environment, combinations of genetic variation that constitutively express the phenotype close to the new optimum would be subject to directional selection.

Cue transfer and genetic adoption each characterize a suite of mechanisms that facilitate the rapid evolution of complex traits by allowing populations to cross fitness valleys rapidly, rather than having to wait for mutations of sufficiently large effect to emerge (1, 33, 34). Importantly, in neither mode is the plastic response to the past cue triggered by the novel cue in the ancestral population, as expected in plasticity-led evolution (5, 6). Therefore, they are neither a form of plasticity-led evolution (6) nor a manifestation of the Baldwin effect (35), but a distinct and potentially underappreciated route for plasticity to influence evolution.

**Fig. 1.**
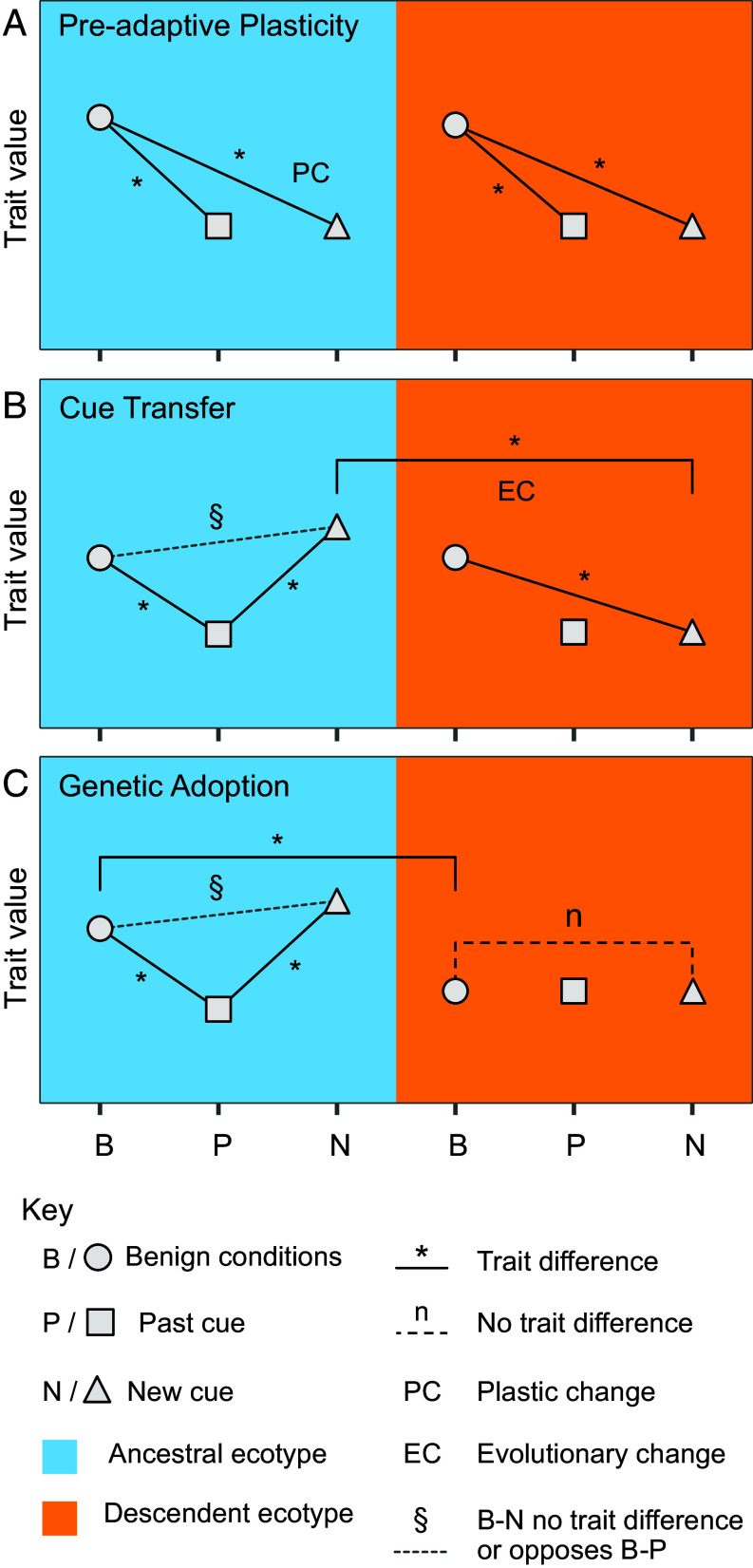
Framework of trait value comparisons for assessing whether plastic responses to past cues are genetically adopted or show cue transfer during adaptation to novel environments. Each panel shows the expected trait value changes in ancestral and descendent environments under different scenarios. Wherever no line is drawn, the trait can take any value. (*A*) Preadaptive plasticity, where ancestral and descendent populations share similar plasticity on exposure to past/new cues. (*B*) Cue transfer, where adaptation results in novel-cue plasticity resembling past-cue plasticity. (*C*) Genetic adoption, where adaptation to the novel environment results in constitutive expression shifting to become more similar to the ancestral population expression value in response to a past cue.

Here, we develop a framework to investigate the role of past-cue plasticity on adaptation. We outline three patterns which point to an influence of past-cue plasticity in the evolution postadaptation trait values: i) preadaptive plasticity—evident as similar phenotypic plasticity in the ancestral and descendent populations in response to both past cues and new cues, without any evidence of an evolutionary change (Fig. 1*A*); ii) cue transfer—characterized by determining whether past-cue plasticity differs from PC and EC moves the descendent population’s plastic response to the new cue toward the past-cue plasticity value rather than to the PC value (Fig. 1*B*); and iii) genetic adoption—identified if past-cue plasticity differs from PC and a constitutive EC during adaptation has taken the trait value closer to the ancestor’s past-cue value (Fig. 1*C*).

Using this framework, we assessed the impact of past-cue gene expression plasticity during parallel adaptive evolution in *Silene uniflora*. In this generally coastal species, several populations have independently colonized and adapted to heavily zinc-contaminated sites at abandoned industrial-era mines (36). Mine populations are locally adapted to this extremely phytotoxic environment, which impacts growth, fitness, and survival of coastal *S. uniflora* (8, 36–38). Mine plants thrive at high zinc concentration and the degree of zinc tolerance for each population is correlated with the level of zinc contamination in local soils (37–39). During adaptation, mine populations have evolved changes in gene expression facilitated by ancestral plasticity to the new environmental cue of high zinc concentrations, characterized by both evolution in the extent of plasticity and genetic assimilation (8, 36). Coastal *S. uniflora* are not exposed to high zinc levels, but they do grow in a challenging saline environment on cliff-tops and rocky shores and exhibit salinity tolerance in seed germination experiments (40, 41). The degree of salt stress in this environment is spatially and temporally variable due to frequent changes in salt deposition rates from sea spray and/or inundation (42, 43). Variability in environmental cues may enhance the evolution of plasticity (44, 45), therefore, we expect a high degree of gene expression plasticity in response to salt exposure in coastal populations. Using coastal populations as a proxy for the ancestors of mine populations, we tested whether gene expression plasticity to a past cue (salt) facilitates adaptation to a new cue (zinc) across two independent evolutionary replicates.

## Results and Discussion

To quantify the extent to which past-cue plasticity influences and is influenced by adaptation to new environments, we sequenced root transcriptomes of individuals from two pairs of coastal and mine-waste-adapted *S. uniflora* populations (Coast-W/Mine-W from Wales, 16.1 km apart, and Coast-E/Mine-E from England, 25.6 km apart) after hydroponic treatment with control and NaCl solutions (*Materials and Methods* and *SI Appendix*, Table S1). Additionally, we reanalyzed transcriptomic data generated from a similar experiment which used the same populations, but grew plants in control and zinc solutions (8). This combination of experiments allowed us to determine; i) the extent to which past-cue plasticity is lost during adaptation to a new cue, ii) the role of preadaptive plasticity in adaptation, and iii) the degree to which plastic responses can switch cues or be genetically adopted during adaptation.

### Adaptation to New Cues Alters the Plasticity Landscape.

**Fig. 2.**
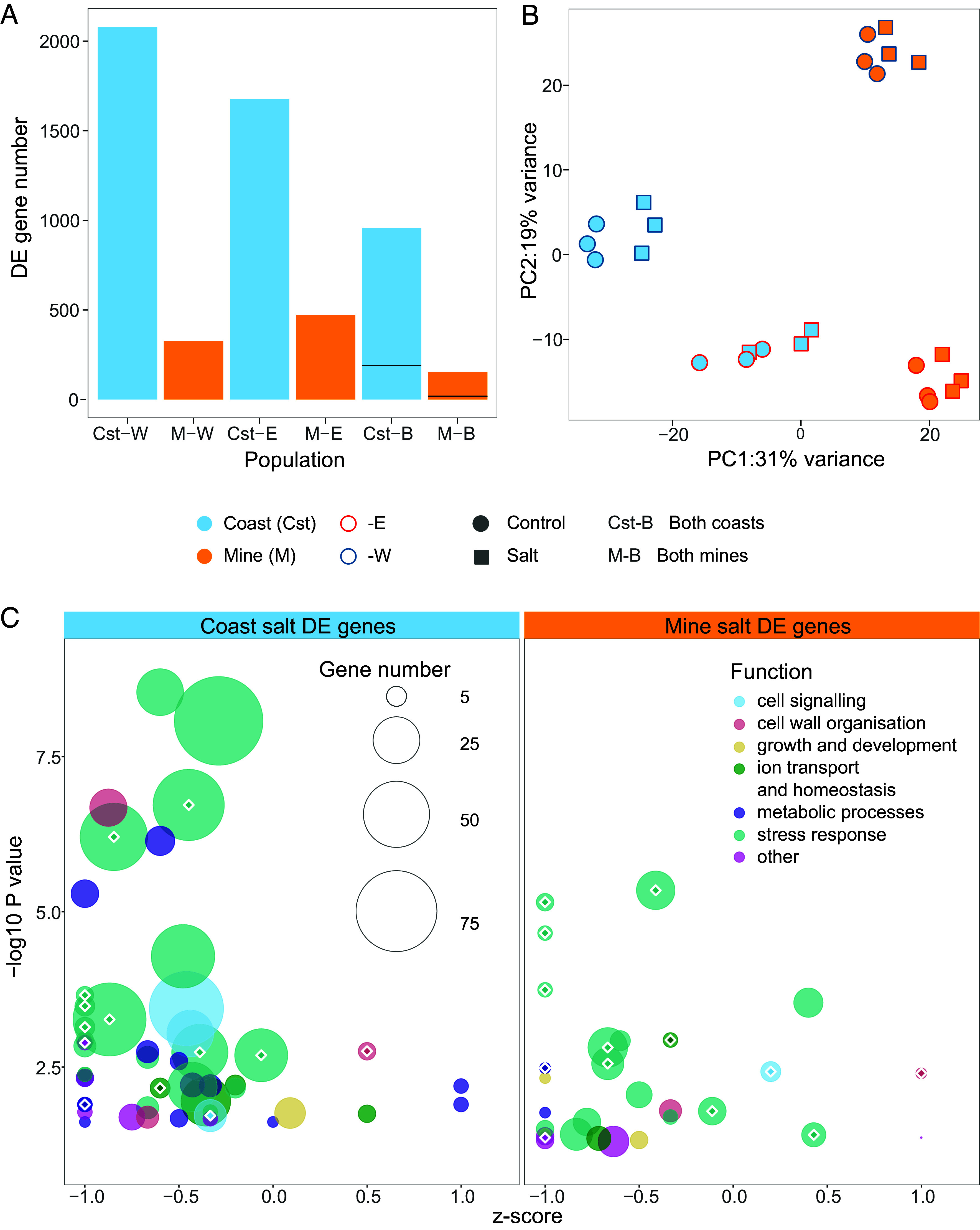
The impact of new-cue (zinc) adaptation on past-cue (salt) plasticity. (*A*) Total number of salt-induced differentially expressed (DE) genes in each population and those genes with the same direction of expression change in both coasts/both mines (Coast-B/Mine-B). The horizontal line denotes the proportion of shred genes expected by chance. (*B*) PCA of variance-stabilized transformed counts of 30,714 genes in each population in control and salt treatments. (*C*) Bubble plot showing the *z*-score of coastal and mine genes differentially expressed in salt within significantly enriched GO categories (*n* up-regulated – *n* down-regulated/total *n* in each GO category) against the negative log-transformed *P*-value for each GO term. 1.0 = 100% up-regulated genes, −1.0 = 100% down-regulated genes, 0.0 = 50% up-/down-regulated genes. Bubbles scale with the number of genes in each GO category and colors represent sets of broader common functions. The 14 common significant GO terms are shown with white outlined diamonds.

We quantified differential expression between coastal and mine populations in response to a past cue (salt) and new cue (zinc) to compare the ancestral and descendent responses to both cues. The coastal populations shared 957 salt-plastic genes with the same direction of expression change (Fig. 2*A*; more than expected by chance: randomization test, *P* < 0.00001; *SI Appendix*, Table S2 and *Methods*), which is roughly half the total number of salt-plastic genes in each individual population (Coast-W = 2,078, Coast-E = 1,676; Fig. 2*A*). Salt plasticity in coastal populations is likely to be adaptive as i) coastal populations display salt tolerance and plasticity in germination under saline conditions (40, 41), ii) there are energetic costs associated with salt-tolerance mechanisms (46, 47), iii) levels of salt stress vary spatially and temporally in this habitat (42, 43) and iv) here, more than 150 of the salt-plastic genes detected in coastal plants had stress-related Gene Ontology (GO) terms (*SI Appendix*, Table S3).

Substantially fewer genes were salt responsive in both mine populations (*n* = 155, Fig. 2*A*; more shared than expected by chance—randomization test, *P* < 0.00001, *SI Appendix*, Table S2). This demonstrates a substantial and parallel loss of plasticity (86.21%) in response to salt stress following adaptation to the mine environment. Although plasticity is reduced, the pattern of expression in response to salt within mine populations resembles that of their coastal ancestors; 85% (*n* = 132) of mine salt-plastic genes were also salt-plastic in both coasts. Upon exposure to salt stress, the proportion of the whole transcriptome that was differentially expressed in both coast/mine populations was quite modest (4.14% for coasts and 0.67% for mines; Fig. 2*B*) when compared to the transcriptome-wide zinc-stress response of coastal populations (47.34%) (8). The breadth of salt-plastic gene functions reduced during adaptation to zinc (36 versus 20 GO terms, *SI Appendix*, Tables S3 and S4), although some functions remain shared between coasts and mines (Fig. 2*C* and *SI Appendix*, Table S5 and Datasets S1 and S2). At the salt concentration used in this experiment, there was no qualitative difference in growth between coastal and mine populations. Environments with consistent, rather than variable, cues are expected to select for reduced plasticity (45). If ancestral salt plasticity is adaptive, exposure to low and stable salt concentrations in the mine environment may have selected for reduced salt plasticity, but some ability to tolerate variable salinity may have been retained despite adaptation to zinc. Such retention of plastic responses to past cues from the ancestral environment may underpin the dominance of plastic changes over genetic adaptations when ancestral environments are recolonized, as found by Ho et al. (48).

**Fig. 3.**
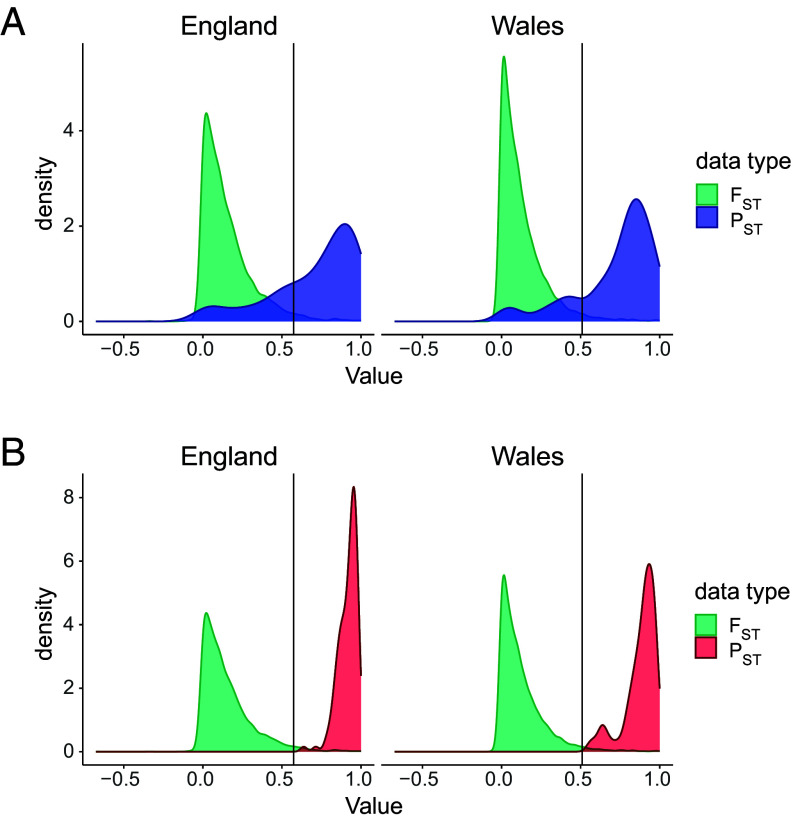
*P_ST_*-*F_ST_* comparisons provide support for adaptive gene expression evolution. *P_ST_* for mine shared zinc-plastic genes (*A*; *n* = 143) and for constitutive expression differences (*B*; *n* = 124) largely exceeds genome-wide *F_ST_* (green) in each population pair.

In line with ref. 8, the reanalyzed zinc experiment included 10,933 zinc-plastic genes shared by both coastal populations (*SI Appendix*, Table S2). The comparison of parallel evolutionary replicates allows us to make the inference that convergent expression patterns in the adapted populations are likely to be the results of selection rather than drift and are at, or close to, the optimum for the new environment (8, 9, 15). In control treatments, 124 genes were differentially expressed between both pairs of coastal and mine populations, showing a pattern of constitutive evolutionary change associated with adaptation. Mine-adapted populations shared 143 zinc-plastic genes (*SI Appendix*, Table S2) with 91 undergoing an evolutionary change in plasticity to zinc (63%). Both gene sets were enriched with functions related to metal tolerance (8) (*SI Appendix*, Tables S6 and S7 and Datasets S3 and S4). This plastic response to zinc is likely to be adaptive, as i) it is extremely convergent in terms of both the genes involved and their expression levels, and ii) it is drastically different to the coastal (largely maladaptive) response. To provide further support that the 124 constitutive and 143 zinc-plastic genes are adaptive, we compared the degree of differentiation in gene expression (*P*_ST_) of these putatively adaptive genes with the underlying neutral distribution of genetic differentiation (*F*_ST_) between mine and coastal populations (15, 49). *P*_ST_ for constitutive differences was calculated from control expressions, whereas the fraction of zinc over control expression was used for zinc-plastic genes. As expected for adaptive expression changes, most genes in these sets have greater *P*_ST_ than expected under neutral differentiation (constitutive differences = 100%; zinc-plastic genes, Wales = 81% and England = 74%; Fig. 3; α = 0.05). Therefore, expression changes for both sets of genes are likely to be adaptive across the independent replicates and they contain proportionally more genes involved in adaptation than the whole transcriptome.

Our focus on parallel expression changes means that genes that are under selection in only one of the lineages are not included in subsequent estimates of the frequency of preadaptive plasticity, cue transfer, and genetic adoption. Ancestral plasticity may increase the chances of a gene being recruited during adaptation across parallel events (8), so it is possible ancestral plasticity is more frequent among parallel adaptive genes than nonparallel adaptive genes. However, divergence due to drift in lineage-specific differentially expressed genes is likely. Further, adaptive genetic variation for specific genes may have only been present in one lineage, which is more probable as zinc tolerance appears to be polygenic (36). It is important to note that in our framework, we can only infer an influence of past-cue plasticity on evolutionary change when the trait optimum in the new environment is similar to the past-cue plasticity value. There are some circumstances where genetically adopted traits would not be detected in our framework; by one mechanism, the ancestral past-cue trait value may not be optimal in the new environment, but ancestral plasticity for that trait can allow greater genetic variation to persist. Additionally, traits for which plastic change is adaptive precludes assessing the potential importance of past-cue plasticity, as do situations where both past-cue and new-cue ancestral responses are maladaptive.

### No Evidence for Preadaptive Plasticity.

To test for evidence of the role of preadaptive plasticity during adaptation to novel stressors, we quantified the number of genes which had significant expression changes in both mine-coast population pairs which were in the same direction in both the control versus salt treatment and between the control versus zinc treatment. We found that there were no genes with this pattern. When the false discovery rate for differential expression analyses was relaxed from 5 to 10%, only one gene followed this pattern (*SI Appendix*, Table S8). This demonstrates that preadaptive plasticity is unlikely to have played a role during adaptation to this novel environment or the signal has been lost very rapidly. Plastic responses to one stressor which are coincidentally also beneficial to another stressor might be expected, as cotolerance has evolved between pressures with similar impacts on plant physiology (23–25) or for chemically similar ions, such as nickel and lead (22) or zinc and nickel/cobalt (21). Both salt and heavy metals produce reactive oxygen species and some molecular mechanisms alleviate the impacts of both stressors (e.g., antioxidants) (23–25). Assuming that salt plasticity in coastal populations is adaptive, this suggests that salt tolerance does not automatically and instantaneously confer zinc tolerance. Some species are known to possess both salt and heavy-metal tolerance in the same population when in habitats with both stressors present (24, 50, 51). Our result suggests that adaptation to both stressors is required in these cases, rather than adaptation to one stressor being preadaptive for the other. The extent of preadaptive plasticity may depend on the similarity (e.g., chemically) between the past and new cues encountered and whether past-cue plasticity actually provides a fitness benefit in the ancestral environment.

### Past-Cue Plasticity Is Transferred to Novel Cues During Adaptation.

**Fig. 4.**
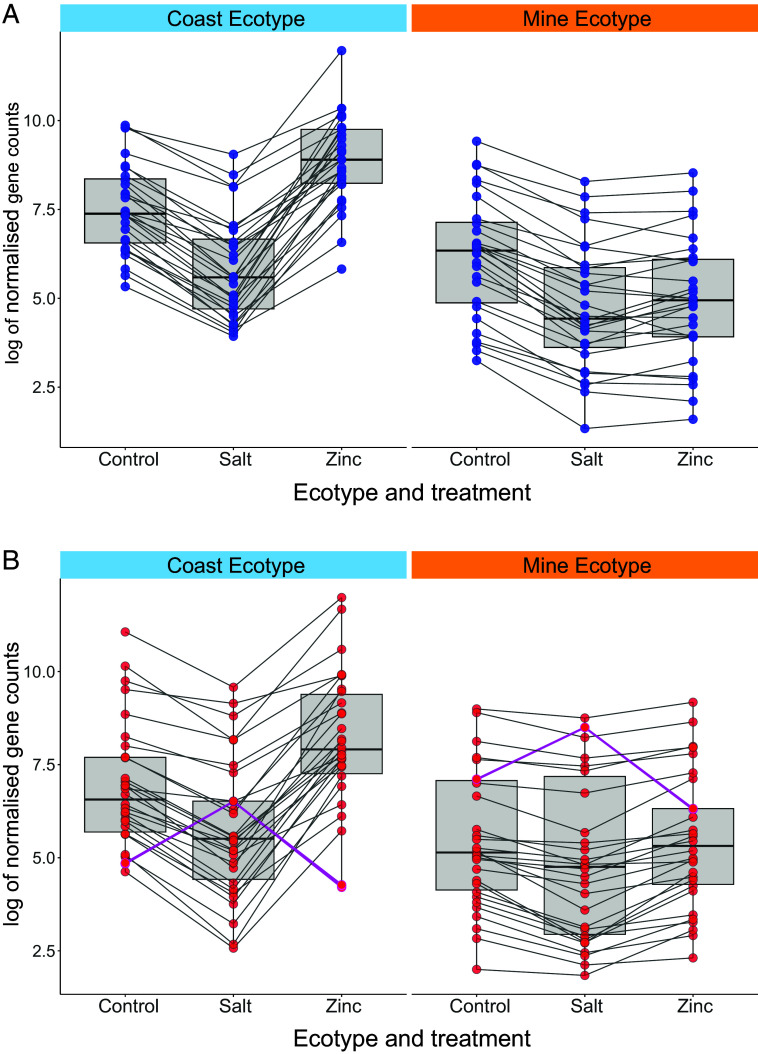
Box and line plots showing the natural log of normalized mean gene expression counts in coast and mine ecotypes (both Welsh and English populations) across control, salt, and zinc treatments for different gene sets. (*A*) Cue transfer genes (*n* = 28) and (*B*) genetically adopted genes (*n* = 30). Points represent individual genes and lines show how mean expression counts differ between each treatment for each gene. Genes for which expression was unregulated in salt are highlighted with magenta lines.

We tested for signals of cue-transfer during adaptation by quantifying the number of coastal salt-plastic genes that underwent a change in zinc plasticity during adaptation *and* for which mine zinc-plasticity matched the direction of the coastal salt response, but not the coastal zinc response. In other words, we looked for genes for which the derived zinc response resembles the ancestral salt response. Cue-transfer occurred for almost one-third of the genes with evolved zinc plasticity (31%, *n* = 28 of 91; Fig. 4*A* and *SI Appendix*, Fig. S1; significantly greater than expected by chance: proportion test, *X*^2^ = 703.82, df = 1, *P*-value < 0.0001; *SI Appendix*, *Methods* and Dataset S5). Cue transfer genes had *P*_ST_-*F*_ST_ signatures consistent with adaptation (*n* Wales = 28 and England = 25; *SI Appendix*, Fig. S2*A*), demonstrating that repurposed past-cue plasticity has played a substantial role in adaptation.

Many molecular pathways are commonly involved in alleviating the consequences of different environmental stresses in different species (23, 24) and so a large component of adaptation may simply be modifying the sensitivity of the pathways to new stressors. Indeed, several of our putative adaptive genes have been implicated in tolerance to both salt and heavy metals, including those involved in signaling pathways (see section below). Based on the potential cue transfer mechanisms described above, we expect that cue transfer is unlikely to operate quickly enough to provide immediate fitness benefits when exposed to a new cue and so cannot be considered as contributing to plasticity-led evolution (6). However, if the population does not die out immediately, cue transfer can provide a route to rapidly crossing fitness valleys by recruiting existing physiological and molecular mechanisms to respond to a new stress which can subsequently undergo adaptive refinement.

Several studies have focused only on changes in plasticity in response to the same cue in ancestral and adapted populations (4, 5, 8–11, 15). Under the frameworks used in these studies, cue transfer genes that are ancestrally plastic to the new cue in the opposite direction to the new optimum (*n* = 17, 61% in this study) would be considered as undergoing reversion. Thus, plasticity in these genes might appear to be maladaptive/nonadaptive in the ancestor if only the new cue is considered. Consequently, by focusing only on the immediate fitness benefit that ancestral plasticity might confer, these studies may underestimate the role that ancestral plasticity has played in increasing the speed and ease of adaptation to new environments.

### Past-Cue Plasticity Is Genetically Adopted During Adaptation.

To provide evidence for genetic adoption, we determined the number of coastal salt-plastic genes with no significant zinc response plasticity in mine plants which did display a constitutive evolutionary change matching the direction of the salt response, but not the zinc response. In other words, we looked for genes for which ancestral plasticity has been lost, but the zinc-adapted trait value is closer to the ancestral salt response. In total, 24% of genes with a constitutive evolutionary change (genes differentially expressed between mine and coastal plants in the control conditions) had expression patterns consistent with genetic adoption (*n* = 30 out of 124; Fig. 4*B*; significantly greater than expected by chance, *X*^2^ = 582.21, df = 1, *P*-value < 0.0001; *SI Appendix*, Fig. S3). All genetically adopted genes had *P_ST_*-*F_ST_* signatures consistent with selection (*SI Appendix*, Fig. S2*B*). During rapid parallel adaptation, genetic adoption occurs almost as frequently as cue transfer.

Previously, Wood et al. (8) found that close to 50% of the genes with constitutive evolutionary changes had undergone genetic assimilation of ancestral zinc plasticity. The genetically adopted genes detected here are mutually exclusive of the genetically assimilated genes, but are from the same larger set (i.e., they all have constitutive evolutionary changes in expression). Taken together, close to three-quarters of constitutive expression changes in *S. uniflora* have been facilitated by ancestral plasticity in response to past (via genetic adoption) or novel cues (via genetic assimilation). Our results show that to understand the role of plasticity more fully during adaptation, it is paramount to test responses to environmental cues found in both ancestral and novel environments.

### Tolerance and Cofunctionality.

We observed a general trend for downregulation among the cue transfer (*n* = 28 of 28) and genetic adoption (n = 27 of 30) genes on exposure to stressors. In part, this is a result of a bias for genes to be down-regulated in response to salt in coastal (*n* up = 259 and down = 700) and mine populations (*n* up = 33 and down = 122). Exclusion is a common route to mitigate ion toxicity, and downregulation of genes that mediate ion transport response to both stressors is possible (38, 46, 52). Similarly, salt and zinc generate reactive oxygen species, and a common solution might be to down-regulate susceptible pathways, such as the mitochondrial electron transport chain and/or photosynthesis (24, 46, 53). Hypermethylation and subsequent downregulation is also a possible mechanism for cue transfer, and this may be a broader characteristic of the influence of past plasticity on adaption.

Although the precise functions of many cue transfer and genetic adoption genes are unknown, several have been implicated in both salt and zinc tolerance (Datasets S6 and S7). Genes encoding chalcone synthase-like proteins (CHS2) were detected among both cue-transfer and genetically adopted genes—chalcone synthase is a key structural enzyme in the flavonoid biosynthetic pathway (54) and has a potential role in the chelation of heavy metals such as copper, lead, cadmium, and nickel (55). Chalcone synthases have also been linked to salinity tolerance (56). Both sets included genes encoding transferases in gene families implicated in salt and heavy-metal stress responses (Datasets S6 and S7) (57–59).

Cue transfer and genetically adopted genes were also enriched for GO terms linked to osmotic, oxidative, and other abiotic stresses linked to heavy-metal responses (*SI Appendix*, Tables S9 and S10). These results support the hypothesis that cofunctionality is present between zinc and salt which may be due to both stressors having overlapping impacts on physiology (23). It may be the case that the more similar the past and novel cue, the more likely it is that past-cue plasticity will influence adaptation. This cofunctionality might explain why we see relatively little indication that there are strong trade-offs between zinc and salt tolerance under these controlled, stable conditions. Most cue transfer genes retained significant salt plasticity in mine populations (Fig. 4*A*) and, unlike the widespread maladaptive transcriptomic response to zinc in coastal plants (8), expression profiles of mine plants did not shift in response to salt any more than the coastal plants. The observed responses under the stable and competition-free conditions of our experiment suggest that many mine adaptations might be conditionally neutral (or even beneficial) in the salt treatment, but these changes could be more disadvantageous in variable, high-competition conditions of wild coastal habitats.

## Conclusion

The role of plasticity in adaptation has become increasingly disputed with evidence both for and against plasticity-led evolution and genetic assimilation. We leveraged instances of parallel adaptation to a recently created novel environment to test the contribution of plasticity to cues from both the new and ancestral environment. Here, we found substantial support for two modes by which past-cue plasticity can facilitate the rapid evolution of complex traits during adaptation to new environments. Overall, three-quarters of the fixed expression differences between ancestral and derived populations can be linked to ancestral plasticity to the past or new cue. Our experiments demonstrate that there is a substantial contribution of ancestral plasticity to both the evolution of new plasticity in expression and canalized expression levels during adaptation.

## Materials and Methods

### Plant Sampling and Hydroponic Experiment.

We studied four populations: Coast-W, Mine-W, Coast-E, and Mine-E corresponding to WWA-C, WWA-M, ENG-C, and ENG-M in ref. 36 and S1, T1, S2, and T2 in ref. 8. An experiment to assess zinc-associated gene expression change was carried out as described in ref. 8. We carried out a near-identical experiment to determine salt (NaCl) associated expression change. Three individuals per population were cloned via mist-propagation and acclimated to deep water hydroponic tanks containing Hoagland’s nutrient solution. After one week, solutions were replaced with either the same solution as a control or the solution plus 0.1 M NaCl (three clones per individual per treatment, *SI Appendix*, Methods). After eight days, root tissue from the clones of each individual was pooled and total RNA was extracted with a Qiagen RNeasy plant kit (*SI Appendix*, *Methods* for more detail). The 24 pools were sequenced on an Illumina NovaSeq platform by Macrogen Genomics Europe. The read length was 100 bp (mean insert size = 101 bp) and the total number of reads per sample was between 40.2 and 43.8 M (*SI Appendix*, Table S11) (60).

### Transcriptome Assembly and Expression Counts.

Raw reads from both the salt and zinc datasets (8) were quality checked and trimmed to remove adapters (see details in *SI Appendix*, *Methods* and Table S11). We used STAR version 2.7.10a (61) to map the trimmed reads to the *S. uniflora* reference genome (*SI Appendix*, *Methods*). The transcriptome was then assembled against the reference genome annotation (62) using StringTie v2.2.0 (63). We generated separate gene expression count matrices for the salt and zinc experiments (41,603 genes in each) using the StringTie prepDE.py3 script (*SI Appendix*, *Methods* and Datasets S8 and S9).

### Differential Expression Analysis.

We used the R package DEseq2 v1.40.0 (64) to analyze the zinc/salt gene expression data (Datasets S8–S10). We filtered both datasets to remove sample counts of <10 and combined them to generate cross-experiment data. To ensure cross-experimental comparability, we filtered all results by genes with no significant differential expression in control conditions between the two experiments, leaving 23,093 genes for further analysis (*SI Appendix*, Fig. S4 and *Methods* for more detail). We conducted principal components analyses with the R package *prcomp* for the salt (30,714 genes, Fig. 2*B*) and combined experiment datasets (30,178 genes, *SI Appendix*, Fig. S5) using variance stabilized transformed counts.

We used two models in *DEseq2* to test for differential expression (α = 0.05) using the input gene expression counts (Datasets S8 and S9) and experimental set-up data (Dataset S10). The first consisted of a single combined factor of *Population + Treatment* to compare within-treatment gene expression between populations and within-population expression between experiments. The second compared within-population gene expression between salt and control or zinc and control treatments, with the formula: *~Population + Population:Individual + Population:Treatment* (details in *SI Appendix*, *Methods*).

### Differential Expression Contrasts for Hypothesis Testing.

We used multiple combinations of differential expression contrasts to determine the impact of novel adaptation on past-cue plasticity and to provide evidence for processes of genetic adoption, cue transfer, and preadaptive plasticity (Fig. 1). Coast or mine salt/zinc plastic genes were those that were differentially expressed between control and salt/zinc treatments in the same direction in both coast or both mine populations. Genes with evolved plasticity to zinc were defined as those differentially expressed between both mine and coastal populations in the zinc treatment and had zinc plasticity in mine populations. Genes with evolved constitutive expression change were defined as those that were differentially expressed between each coast and mine in control conditions as in ref. 8. *P*_ST_ for expression levels in control and the fraction of zinc over control expression was calculated using the R package *pstat* and compared to the genome-wide distribution of *F*_ST_ from (36) (Fig. 3 and *SI Appendix*, *Methods*).

To test for preadaptive plasticity, we searched for genes with the same plasticity to salt/zinc and shared salt and zinc plasticity in coastal plants (*SI Appendix*, Fig. S6*A*). To test for cue transfer, we identified the coastal salt-plastic genes for which ancestral salt and zinc responses were not the same and which had evolved plasticity to zinc in the mine populations matching ancestral salt plasticity (*SI Appendix*, Fig. S6*B*). To test for genetic adoption, we searched for genes involved in coastal responses to salt that also had matching constitutive evolved expression changes, were not differentially expressed between control and zinc treatment in mines, and for which ancestral salt and zinc responses differed (*SI Appendix*, Fig. S6*C*). *SI Appendix*, *Methods* for more details.

### Functional Analyses.

The function of genes within the sets of interest was determined using the *S. uniflora* reference annotation (62). We also conducted GO enrichment using topGO v2.52.0 (*SI Appendix*, *Methods* for more details).

## Supplementary Material

Appendix 01 (PDF)

Dataset S01 (XLSX)

Dataset S02 (XLSX)

Dataset S03 (XLSX)

Dataset S04 (XLSX)

Dataset S05 (XLSX)

Dataset S06 (XLSX)

Dataset S07 (XLSX)

Dataset S08 (CSV)

Dataset S09 (CSV)

Dataset S10 (XLSX)

## Data Availability

Raw sequencing reads data have been deposited in SRA (PRJNA1113995) (60).
