# Supplementary material for: Plastic responses to past environments shape adaptation to novel selection pressures
Source: Proc Natl Acad Sci U S A. 2025 Jan 30;122(5):e2409541122. doi: 10.1073/pnas.2409541122 (PMC11804578; doi:10.1073/pnas.2409541122)
Supplement: Supplementary file 1 — Appendix 01 (PDF) [file pnas.2409541122.sapp.pdf]

PNAS

Supporting Information for

Plastic responses to past environments shape adaptation to novel selection pressures

Sarah E. R. Coates[1,2], Aaron A. Comeault[1], Daniel P. Wood[2], Michael F. Fay[2,3], Simon Creer[1], Owen G. Osborne[1], Luke T Dunning[4], Alexander S. T. Papadopulos[1]

1. Molecular Ecology and Evolution group, School of Environmental and Natural Sciences, Bangor University, Bangor, UK. 2. Royal Botanic Gardens Kew, Richmond, UK. 3. School of Plant Biology, University of Western Australia, Crawley, WA 6009, Australia. 4. Ecology & Evolutionary Biology, School of Biosciences, University of Sheffield, Sheffield, UK.

Corresponding Author : Alexander S.T. Papadopulos
Email: a.papadopulos@bangor.ac.uk

This PDF file includes:

Supplementary Methods
Figures S1 to S6
Tables S1 to S11
Legends for Datasets S1 to S10
SI References

Other supporting materials for this manuscript include the following:

Datasets S1 to S10

Supplementary Methods

Experimental design and hydroponics

To analyse salt associated gene expression changes, we used three individuals from each of four populations (Table S1). The individuals were either from existing *S. uniflora* living collections (*n* = 4, germinated as in Wood *et al.* (1)) or more recently germinated individuals (*n* = 8). All samples were grown from seed collected as part of a previous study (2) - population codes in this study: Coast-W, Mine-W, Coast-E, Mine-E; correspond to WWA-C, WWA-M, ENG-C and ENG-M in Papadopulos *et al.* (2); and S1, T1, S2, T2 in Wood *et al.* (1), respectively. One Mine-W individual and one Mine-E individual were the same plant as sampled in Wood *et al.* (1). Newly germinated plants were transferred to 1.5L pots three months after germination and grown on under ambient conditions for c. eight months. Cuttings were taken from three individuals per population and were rooted using mist propagation for two weeks with 12L diH_2O and a further two weeks with 12L diH_2O containing 1.92g Hoagland's nutrient powder. After four weeks of propagation, an equal proportion of cuttings from each population (*n* = 6) were transferred to six deep water hydroponic growth tanks filled with 0.16%w/v Hoagland's nutrient solution and allowed to acclimate for one week. The Hoagland's nutrient solution was made up by dissolving 1.28g of Hoagland's number two powder in 0.8ml diH_2O per tank and adding KOH to adjust the pH to 5.5. The solution was diluted in each tank by adding 7.2L diH_2O per tank, taking the total volume per tank to 8L. After acclimation, three tanks received fresh Hoagland's solution alone and three tanks received 0.1M NaCl dissolved in 0.16%w/v Hoagland's nutrient solution (three clones per individual in each treatment). Similar concentrations of NaCl elicit changes in phenotype in plant species such as other *Silene* (3, 4) and the halophyte *Armeria maritima* (5, 6). After eight days of these treatments, root tissue from the clones of each individual were pooled within treatments, flash frozen in liquid nitrogen and stored at -80°C. Pools of root tissue were homogenised and total RNA was extracted from 70-100mg tissue using a Qiagen RNeasy plant kit with an elution volume of 50µl. Tissue disruption with stainless steel beads and sand was carried out via the Tissuelyser Lt machine with a cooled head at 50Hz for two minutes. RNA sequencing (100bp, paired end) of the resulting 24 root samples (three individuals per population) was carried out using TruSeq stranded mRNA (llumina) library preps and run on an Illumina Novaseq by Macrogen Genomics Europe. The total number of reads (paired, forward and reverse) was between 40.2 and 43.8 million per sample (Table S11).

Read processing and mapping

Quality control information was generated with FastQC (7) and summarised using multiQC software (8). Reads were trimmed and filtered using Trimmomatic, with the options of: -phred33 ILLUMINACLIP:${MYADAPS}:2:30:10 LEADING:3 TRAILING:3 SLIDINGWINDOW:4:10 MINLEN:50 (9). MYADAPS='/apps/genomics/trimmomatic/0.39/adapters/TruSeq3-PE-2.fa'. The reference genome *S. uniflora* (10) was indexed, and reads of each individual sample mapped to the reference genome using STAR v2.7.10a (11). To complete the mapping step, STAR v2.7.10a (11) was run with the following options: "outSAMtype BAM SortedByCoordinate; outSAMstrandField intronMotif; outSAMattributes NH HI AS nM XS". Reference-based transcriptome assembly was carried out in StringTie v2.2.0 (12) using the reference annotations for *S. uniflora* and the following options: -e -B -p 10. We used the transcriptome assembly to generate a matrix of gene counts for 41,603 genes for both salt and zinc experiments in the format for downstream analysis by running the StringTie python script, *prepDE.py3*, with the -g option (Datasets S8 & S9).

Differential expression analyses

We used the R package *DEseq2* v1.40.0 (13) to analyse our gene expression count data and test for differential expression, at an alpha level of 0.05. We filtered the zinc and salt count datasets to remove sample counts of <10 and combined them to generate cross-experiment expression data. We conducted principal components analyses with the R *prcomp* function for the salt experiment alone (30,714 genes, Figure 2B) and for the experiments combined (30,178 genes, Figure S5) using variance stabilised transformed counts. The control treatments for both salt and zinc experiments were tightly clustered in the PCA (Figure S5). This suggests the two separate

experiments were highly consistent and, thus, could be combined. To ensure comparability between the experiments for expression comparisons, we created a set of genes that were not differentially expressed between the control conditions for the two experiments (Figure S4). Only genes for each population that were not differentially expressed between controls were included. After this filtering, 23,093 genes were available for further analysis (~56% of the total number assembled).

We analysed the three filtered expression matrices (see Datasets S8 and S9 for unfiltered matrices) with the corresponding experimental structures (Dataset S10), using *DeSeq2's* built-in models to identify genes that were significantly differentially expressed (with a p value below 0.05). We applied two differential expression models to quantify differential expression in three ways: (i) between population differential expression within treatments (e.g. Coast-W control vs Mine-W control), (ii) between experiment and treatment conditions within populations (e.g. Coast-W salt experiment control vs Coast-W zinc experiment control and salt vs zinc comparisons); and (iii) between treatments, within populations and experiments (e.g. control vs salt in Coast-W). The first model to test (i) and (ii) consisted of a single combined factor of *Population+Treatment*. The second model examined (iii) using the formula: ~ *Population* + *Population:Individual* + *Population:Treatment*. These models were also repeated using a significance threshold of 0.1, for a comparison of results of different stringencies to see if consistent patterns of gene expression change remained.

Framework of differential expression contrasts for hypothesis testing

We used multiple combinations of differential expression comparisons to determine the impact of novel adaptation on past-cue plasticity and to provide evidence for processes of genetic adoption, cue transfer and pre-adaptive plasticity (Figure 1). We first categorised genes into distinct groups. Coast or mine salt/zinc plastic genes were those that were differentially expressed between control and salt/zinc treatment in the same direction in both coast, or in both mine populations (Table S2). The 91 genes that have newly evolved plasticity to zinc were those that were differentially expressed between both mine and coastal populations in the zinc treatment, *and* between control and zinc in mine populations. The 124 Genes that had evolved constitutive expression changes were defined as those that were differentially expressed in the same way between each coast and mine in the control conditions as in (1).

To test for pre-adaptive plasticity, we searched for genes with shared mine salt and zinc plasticity (i.e., mine responses to both treatments were the same), *and* shared salt and zinc plasticity in coastal plants (Figure S6A). To test for cue transfer, we identified the coastal salt-plastic genes that were differentially expressed between salt and zinc conditions in coastal plants, had no initial zinc plasticity or a different direction of differential expression from control to zinc versus control to salt (i.e., ancestral salt and zinc responses were not the same) *and* had evolved shared plasticity to zinc in the mine populations in the same direction as the ancestral salt response (Figure S6B). To test for genetic adoption, we searched for coastal salt-plastic genes with matching evolved constitutive expression changes, that had a different ancestral plasticity response in control/salt versus control/zinc, were differentially expressed between salt and zinc in both coasts and were *not* differentially expressed between control and zinc treatment in mines (Figure S6C).

The significance of the number of differentially expressed genes in response to salt and zinc shared across population pairs was analysed using a randomisation test. The test consisted of 10,000 replications of random draws of differentially expressed genes from the total number of filtered genes (23,093). An empirical p-value was calculated for each randomisation test, which was calculated as the frequency of randomisations producing overlaps that were more than or equal to the observed number divided by the number of replications. We also determined the maximum overlap for each randomisation for comparison with the observed overlap (Table S2).

We used a proportion test to determine if the observed proportion of cue transfer and genetically adopted genes was higher than expected by chance. We determined the expected proportions from the total number of possible alternative patterns that could be observed based on the criteria applied. In a single population, two out of 19 possible outcomes fit the expectations for cue transfer and genetic adoption (Dataset S5). Under the assumption that all possible patterns are equally likely, the probability that two independent pairs share these patterns is 0.011 (2/19*2/19).

We determined the phenotypic differentiation (P_{ST}) for expression phenotypes from genes likely to be involved in parallel adaptation to the mine environment and compared this to genomic differentiation (F_{ST}). P_{ST} between each mine and coast was calculated for each gene of interest for the genes with mine zinc plasticity (n = 143, Figure 3A) and the genes with evolved constitutive changes (n = 124, Figure 3B). using the R package *pstat* (16). P_{ST} for mine zinc-plastic genes was calculated using the phenotype of the fraction of zinc/control expression, whereas the constitutively evolved gene phenotype was their expression level in control treatments. Genomic differentiation values between Welsh coast versus mine and English coast versus mine populations were taken from results from the dataset analysed by Papadopulos *et al.* (2). Genes were defined as those likely to be under selection as those with P_{ST} values higher than the top 5% of all genomic F_{ST} values. We also calculated P_{ST} between each mine and coast population for the genes meeting patterns of cue transfer and genetic adoption to compare to genomic F_{ST} as shown in Figure S2. The phenotypes used were the fraction of zinc/control expression for cue transfer and the control expression levels for genetic adoption.

We compared the number of genes likely to be involved in adaptation and with pre-adaptive plasticity, cue transfer and genetic adoption patterns determined with an alpha of 0.05 (results reported in the main text and supporting information) vs 0.1. The two sets of results are comparable to each other, as shown in Table S8.

Functional analyses

We determined the function of genes within the sets of interest using *Silene uniflora* reference annotations (10). These sets included those genes involved in cue transfer and genetic adoption (Datasets S6 & S7), as well as the mine zinc-plastic genes and constitutively evolved genes (Datasets S3-4). We conducted GO ontology enrichment to detect functional enrichment within our gene sets of interest using *topGO* v2.52.0 (14). We conducted this analysis on genes plastic to salt in both coasts and mines (Tables S3-S5), genes involved in constitutive adaptation and mine-zinc plastic genes (Tables S6 & S7) as well as those for cue transfer and genetically adopted gene sets (Tables S9 & S10). To determine how the enriched GO terms differed between mines and coasts in salt plastic genes, we determined the *z*-score (z = sum(+ve LFC genes) – sum(-ve LFC genes)/ total n of genes in GO category) for each gene within a significant enriched GO (Figure 2C). We based this calculation from the *z*-score calculation described in the R *GOplot* package (15). We highlighted GOs by broader shared functional categories that were defined based on the descriptions of the GO terms (Figure 2C, Tables S3-5). This gives an indication of the patterns of expression change within the enriched GO term. We also looked for the ontology of genes likely show zinc adaptation; those with evolved constitutive change and the mine shared zinc-plasticity genes (Tables S6 & S7), For the sets of genes with cue-transfer and genetic adoption, we searched for enriched GO terms linked to salt/osmotic stress and heavy metal stress to find co-functionality between these stress responses (Tables S9 & S10). We also studied the functional annotations themselves to look for genes with potential salt and heavy metal stress co-functionality (Datasets S6 & S7).

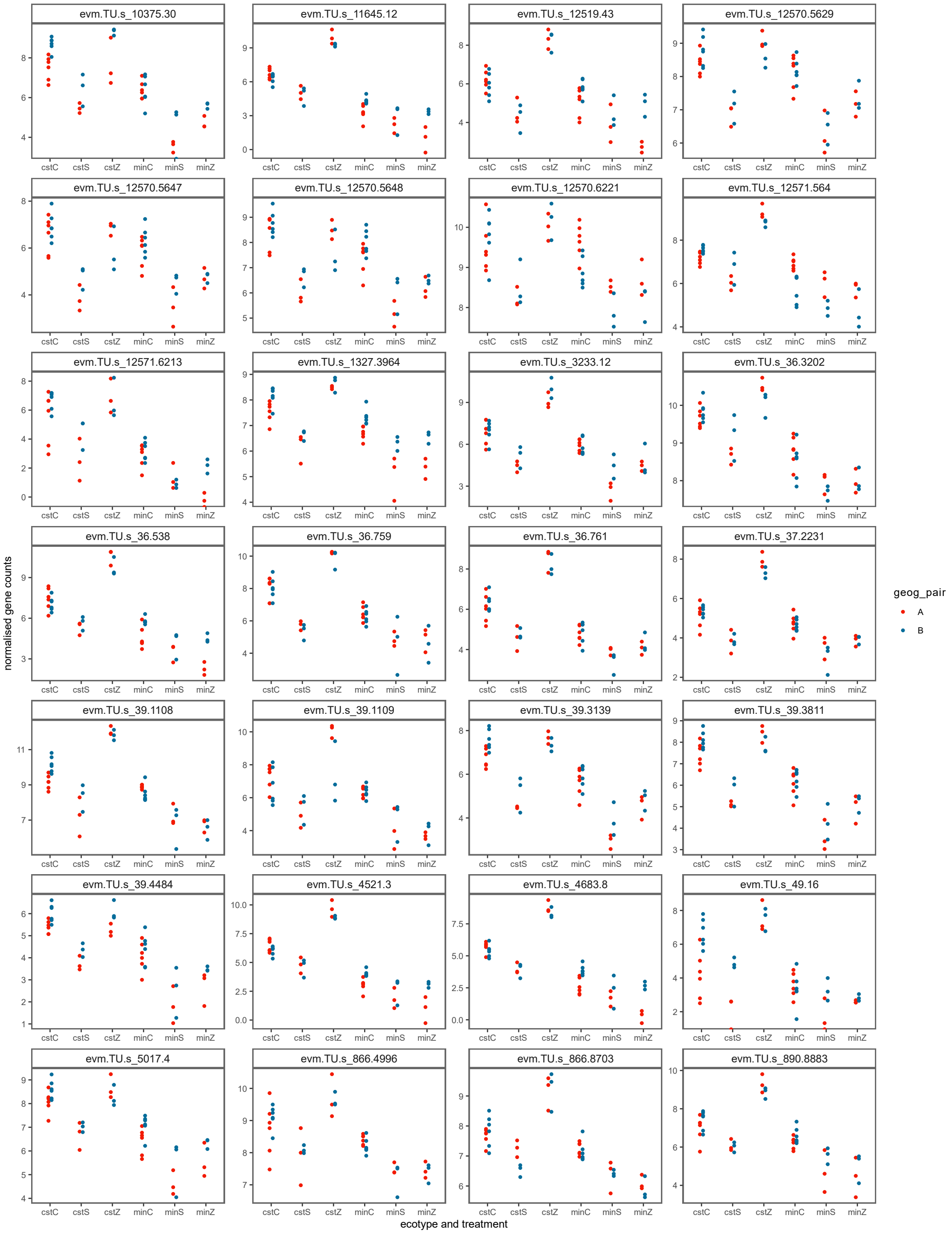

Figure S1. Gene by gene expression patterns for 28 Cue transfer genes. Normalized expression counts across all 6 ecotype-treatment combinations for each gene with evidence of genetic adoption. Each point represents a sequenced individual and the different colours represent each population studied. The control treatments contain 6 individuals per population as zinc/salt experimental samples have been combined and the zinc and salt treatments have 3 individuals per population.

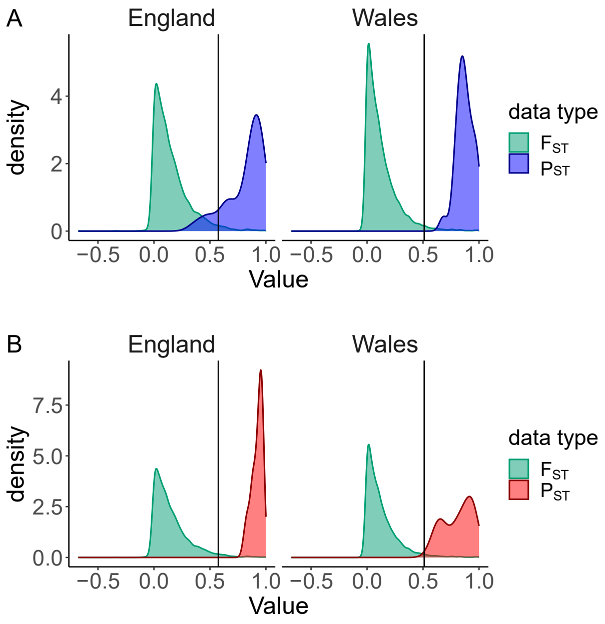

Figure S2. P_{ST} of gene expression phenotypes for (A) cue transfer and (B) genetically adopted genes versus F_{ST} across the *S. uniflora* genome from RAD sequence data generate by (2). The phenotype used for cue transfer was the fold change of Z/C in each coast and each mine and the phenotype for genetic adoption was the normalised expression counts in control conditions in each coast and each mine population. All genes had P_{ST} values within the top 5% of outlier values for the genome, except for three cue transfer genes in England that were within the top 20% of outlier values.

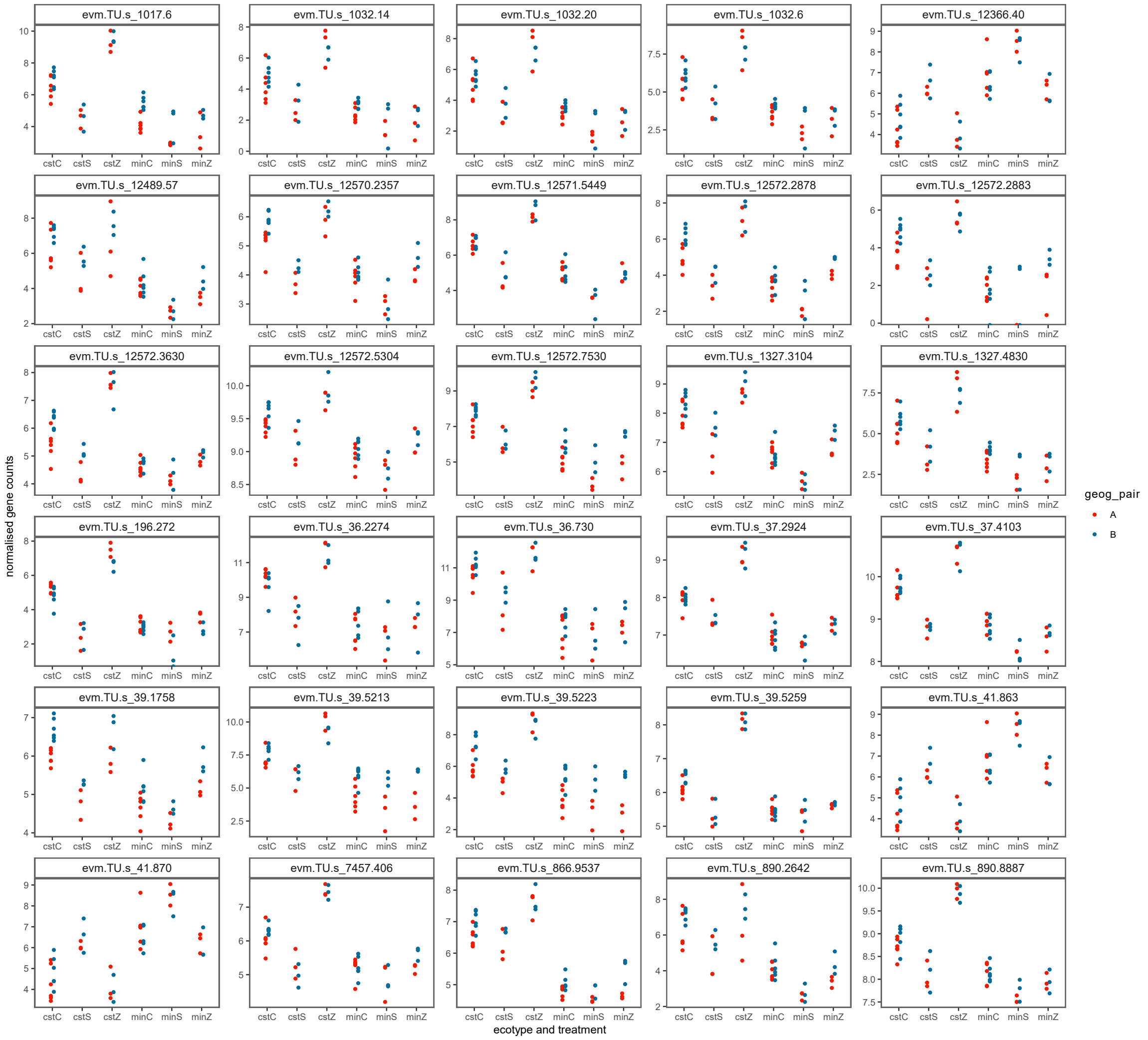

Figure S3. Gene by gene expression patterns for 30 genetically adopted genes. Normalized expression counts across all 6 ecotype-treatment combinations for each gene with evidence of genetic adoption. Each point represents a sequenced individual and the different colours represent each population studied. The control treatments contain 6 individuals per population as zinc/salt experimental samples have been combined and the zinc and salt treatments have 3 individuals per population.

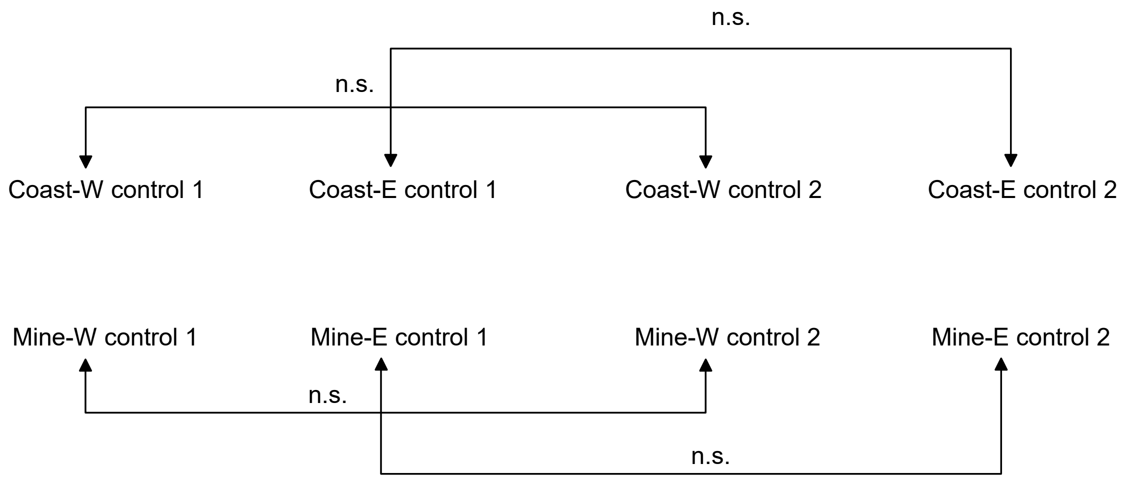

Figure S4. Set of differential expression contrasts used for ensuring cross-experimental comparability (n = 23,093). n.s.= no significant differential expression, Salt experiment = 1, Zinc experiment = 2.

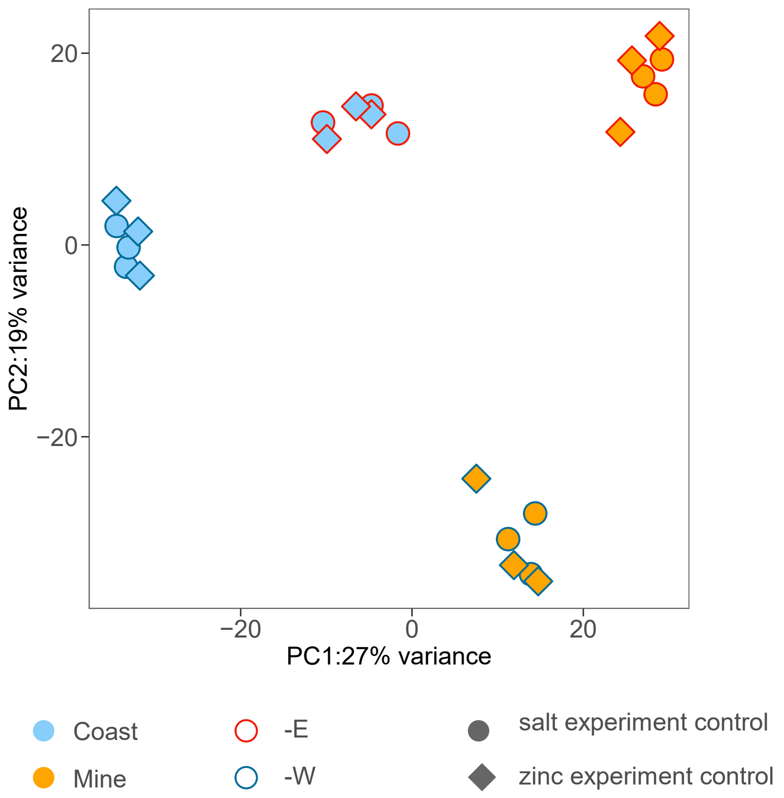

Figure S5. Principle components 1 and 2 from PCA of control treatments from salt and zinc experiments.

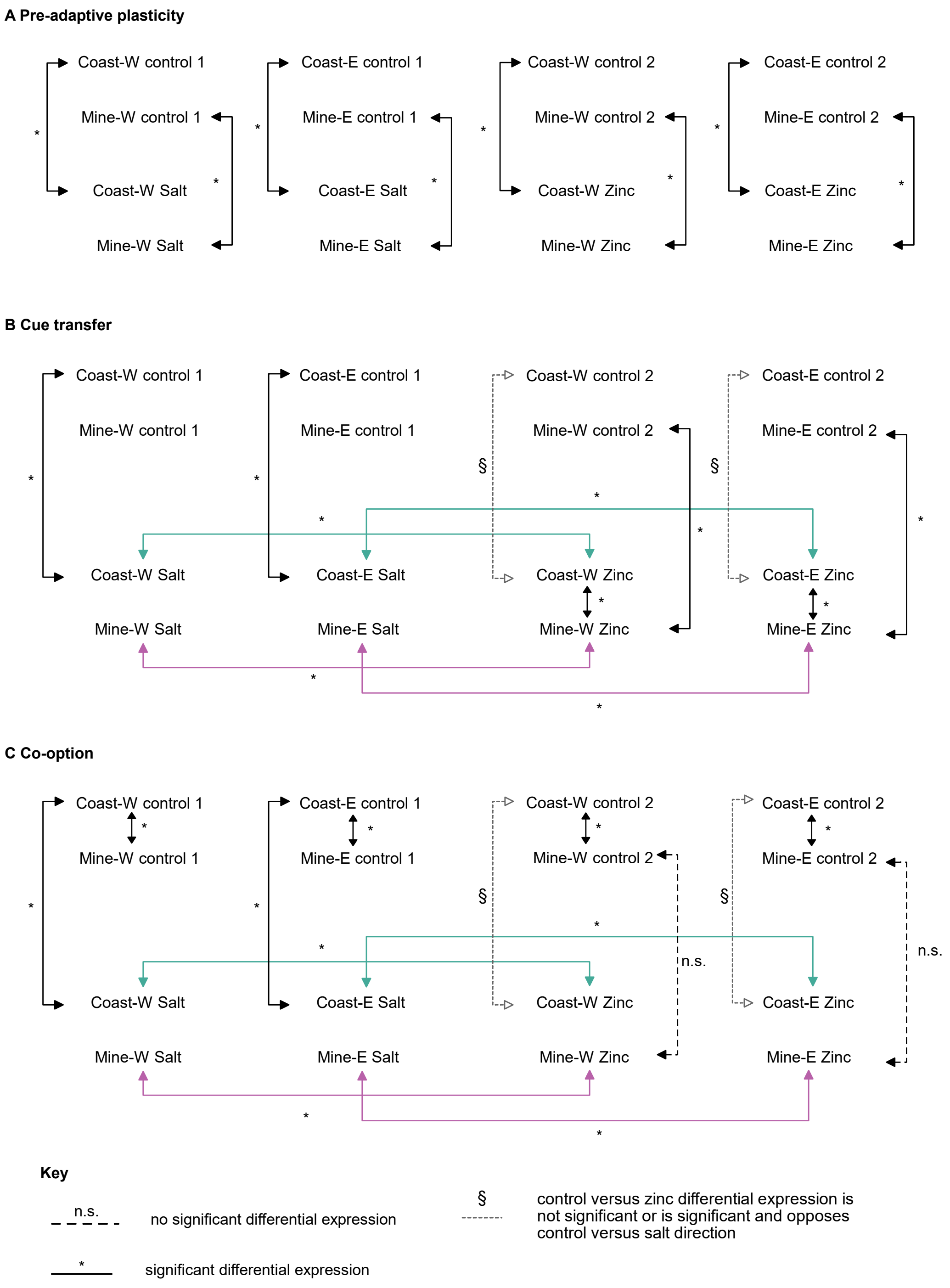

Figure S6. Differential expression comparisons network for (A) pre-adaptive plasticity (*n* = 0), (B) cue transfer (*n* = 28) and (C) genetic adoption (*n* = 30). Shared line colours to mark the contrasts denote the sets that were filtered to those only with the same direction of expression change across the set.

Table S1. Population coordinates and locations for sampled *Silene uniflora* populations. Included are the population names used in both Wood *et al.* (1) and Papadopulos *et al.* (2) The two letter population codes used in sequencing are also listed.

Population name	Name in Papadopulos *et al.* 2021	Name in wood *et al.* 2023	Latitude	Longitude	Country	Location	Population code for sequencing
Coast-W	WWA-C	S1	52.394825	-4.0939136	Wales	Aberystwyth	SA
Mine-W	WWA-M	T1	52.331608	-3.887207	Wales	Grogwynion	GR
Coast-E	ENG-C	S2	51.323284	-3.0169961	England	Brean Down	BD
Mine-E	ENG-M	T2	51.256935	-2.6509495	England	Priddy Pools	PP

Table S2. Sets of shared salt/zinc plastic genes between mines and coasts and randomization tests. size set 1 = Welsh population number of Differentially expressed genes control to salt/zinc; size set 2 = English population number of differentially expressed genes control to salt/zinc. Observed overlap = actual number of shared genes. Maximum randomised overlap = maximum number of shared genes from 10,000 randomisations. Empirical *p*-value = frequency of observed overlap from 10,000 randomisations.

Gene set of interest	Size set 1	Size set 2	Observed overlap	Maximum randomised overlap	Empirical *p*-value
Coastal shared salt plasticity	2078	1676	957	191	<0.0001
Mine shared salt plasticity	326	472	155	18	<0.0001
Coastal shared zinc plasticity	13461	13343	10933	7918	<0.0001
Mine shared zinc plasticity	241	836	143	23	<0.0001

Table S3. Significantly enriched GO terms for shared Coastal salt-plastic genes only (number of terms = 36), with broader functional classification.

ID	Description	Broader function	Gene number in GO	*p*-value
GO:0010200	response to chitin	stress response	25	2.90E-09
GO:0001101	response to acid chemical	stress response	90	8.40E-09
GO:0009808	lignin metabolic process	cell wall organisation	16	2.10E-07
GO:0120256	olefinic compound catabolic process	metabolic processes	10	7.20E-07
GO:0006568	tryptophan metabolic process	metabolic processes	9	5.10E-06
GO:0097305	response to alcohol	stress response	46	5.20E-05
GO:0033993	response to lipid	cell signalling	63	0.00036
GO:0042445	hormone metabolic process	cell signalling	25	0.00091
GO:0060992	response to fungicide	stress response	4	0.00117
GO:0009696	salicylic acid metabolic process	stress response	6	0.00144
GO:0046351	disaccharide biosynthetic process	metabolic processes	6	0.00179
GO:0009407	toxin catabolic process	stress response	6	0.0022
GO:0046355	mannan catabolic process	metabolic processes	4	0.00254
GO:0006032	chitin catabolic process	stress response	3	0.00415
GO:0043312	neutrophil degranulation	stress response	3	0.00415
GO:0019762	glucosinolate catabolic process	metabolic processes	4	0.00472
GO:0032527	protein exit from endoplasmic reticulum	other	4	0.00472
GO:0015718	monocarboxylic acid transport	metabolic processes	7	0.00614
GO:0006879	intracellular iron ion homeostasis	ion transport and homeostasis	5	0.00615
GO:0042759	long-chain fatty acid biosynthetic process	metabolic processes	6	0.00632
GO:0006012	galactose metabolic process	metabolic processes	3	0.00639
GO:0046686	response to cadmium ion	stress response	28	0.00663
GO:0017001	antibiotic catabolic process	stress response	5	0.00688
GO:0006820	monoatomic anion transport	ion transport and homeostasis	28	0.01125
GO:0005978	glycogen biosynthetic process	metabolic processes	3	0.01267

GO:0006749	glutathione metabolic process	stress response	6	0.01434
GO:0045332	phospholipid translocation	other	3	0.01675
GO:2000379	positive regulation of reactive oxygen species metabolic process	stress response	3	0.01675
GO:0009965	leaf morphogenesis	growth and development	11	0.01738
GO:0055074	calcium ion homeostasis	ion transport and homeostasis	4	0.01786
GO:0018105	peptidyl-serine phosphorylation	other	8	0.0202
GO:0052545	callose localization	cell wall organisation	6	0.02025
GO:0046890	regulation of lipid biosynthetic process	metabolic processes	4	0.02118
GO:2000117	negative regulation of cysteine-type endopeptidase activity	other	3	0.02149
GO:0000023	maltose metabolic process	metabolic processes	2	0.02419
GO:0009805	coumarin biosynthetic process	metabolic processes	2	0.02419

Table S4. Significantly enriched GO terms for shared mine salt-plastic genes only (number of terms = 20), with broader functions classified.

ID	Description	Broader function	Gene number in GO	*p*-value
GO:0009414	response to water deprivation	stress response	10	0.00029
GO:0009615	response to virus	stress response	5	0.00119
GO:0048768	root hair cell tip growth	growth and development	2	0.00476
GO:0009620	response to fungus	stress response	8	0.00883
GO:0009664	plant-type cell wall organization	cell wall organisation	6	0.01598
GO:0010192	mucilage biosynthetic process	metabolic processes	2	0.01714
GO:0010119	regulation of stomatal movement	stress response	3	0.02015
GO:0002376	immune system process	stress response	9	0.0242
GO:0098869	cellular oxidant detoxification	stress response	4	0.03157
GO:0010214	seed coat development	growth and development	2	0.03768
GO:0009636	response to toxic substance	stress response	12	0.03823
GO:0007568	aging	other	4	0.0411
GO:0007187	G protein-coupled receptor signaling pathway, coupled to cyclic nucleotide second messenger	cell signalling	1	0.04321
GO:0007188	adenylate cyclase-modulating G protein-coupled receptor signaling pathway	cell signalling	1	0.04321
GO:0035461	vitamin transmembrane transport	other	1	0.04321
GO:0071786	endoplasmic reticulum tubular network organization	other	1	0.04321
GO:0098656	monoatomic anion transmembrane transport	ion transport and homeostasis	7	0.04453
GO:0009860	pollen tube growth	growth and development	4	0.04734
GO:0010150	leaf senescence	other	4	0.04734
GO:0006468	protein phosphorylation	other	11	0.04992

Table S5. Significantly enriched GO terms shared across both coastal and mine ecotype (number of terms = 14), with broader functions classified.

ID	Description	Broader function	Coastal gene number in GO	Mine gene number in GO	Coastal *p*-value	Mine *p*-value
GO:0006970	response to osmotic stress	stress response	58	17	1.90E-07	4.50E-06
GO:0009617	response to bacterium	stress response	52	12	6.20E-07	0.00278
GO:0072722	response to amitrole	stress response	4	3	0.00022	2.20E-05
GO:0010272	response to silver ion	stress response	5	3	0.00033	0.00018
GO:0098542	defense response to other organism	stress response	61	18	0.00054	0.00153
GO:0009635	response to herbicide	stress response	5	4	0.00072	7.00E-06
GO:0016598	protein arginylation	other	3	1	0.00128	0.04321
GO:0009828	plant-type cell wall loosening	cell wall organisation	4	2	0.00176	0.00399
GO:0006979	response to oxidative stress	stress response	36	9	0.00183	0.0162
GO:0009409	response to cold	stress response	32	7	0.00204	0.03899
GO:0032412	regulation of monoatomic ion transmembrane transporter activity	ion transport and homeostasis	5	3	0.00688	0.00116
GO:0010392	galactoglucomannan metabolic process	metabolic processes	3	2	0.01267	0.00328
GO:0051070	galactomannan biosynthetic process	metabolic processes	3	2	0.01267	0.00328
GO:0080167	response to karrikin	cell signalling	12	5	0.01913	0.00374

Table S6. Significantly enriched GO terms for 124 genes with constitutive expression changes between both coast and mine populations (number of terms = 35).

ID	Description	Gene number in GO	*p*-value
GO:0006970	response to osmotic stress	11	9.90E-06
GO:0010726	positive regulation of hydrogen peroxide metabolic process	2	0.00035
GO:0009875	pollen-pistil interaction	2	0.00407
GO:1900055	regulation of leaf senescence	2	0.006
GO:0009414	response to water deprivation	6	0.00629
GO:0010043	response to zinc ion	2	0.01317
GO:0010942	positive regulation of cell death	2	0.01317
GO:2000024	regulation of leaf development	2	0.01377
GO:0010200	response to chitin	3	0.01442
GO:0002229	defense response to oomycetes	2	0.01975
GO:0042304	regulation of fatty acid biosynthetic process	1	0.02071
GO:0016598	protein arginylation	1	0.02071
GO:0006911	phagocytosis, engulfment	1	0.02071
GO:0042659	regulation of cell fate specification	2	0.02347
GO:0090708	specification of plant organ axis polarity	2	0.02424
GO:0061088	regulation of sequestering of zinc ion	1	0.02481
GO:0042149	cellular response to glucose starvation	1	0.02481
GO:2000067	regulation of root morphogenesis	2	0.02663
GO:0048316	seed development	6	0.02717
GO:0001508	action potential	1	0.03294
GO:0046244	salicylic acid catabolic process	1	0.03294
GO:0043434	response to peptide hormone	1	0.03294
GO:0015700	arsenite transport	1	0.04101
GO:0046898	response to cycloheximide	1	0.04101
GO:0009704	de-etiolation	1	0.04101
GO:0019288	isopentenyl diphosphate biosynthetic process, methylerythritol 4-phosphate pathway	1	0.04101
GO:0007265	Ras protein signal transduction	1	0.04101
GO:0060992	response to fungicide	1	0.04101
GO:0009820	alkaloid metabolic process	1	0.04502
GO:1902418	(+)-abscisic acid D-glucopyranosyl ester transmembrane transport	1	0.04502
GO:0071236	cellular response to antibiotic	2	0.04882
GO:0006457	protein folding	2	0.04882
GO:0046355	mannan catabolic process	1	0.04901
GO:0010205	photoinhibition	1	0.04901

Table S7. Significantly enriched GO terms for 143 genes with zinc-plasticity shared between both mine populations (number of terms = 39).

ID	Description	Gene number in GO	*p*-value
GO:0072722	response to amitrole	5	2.00E-10
GO:0009635	response to herbicide	6	2.70E-10
GO:0010272	response to silver ion	5	1.20E-08
GO:0009620	response to fungus	10	4.90E-05
GO:0055062	phosphate ion homeostasis	3	7.10E-05
GO:0009627	systemic acquired resistance	5	7.40E-05
GO:0009615	response to virus	5	0.00029
GO:0007568	aging	6	0.00037
GO:0009407	toxin catabolic process	3	0.00068
GO:0006032	chitin catabolic process	2	0.00085
GO:0043312	neutrophil degranulation	2	0.00085
GO:0055081	monoatomic anion homeostasis	3	0.00112
GO:0051707	response to other organism	19	0.00118
GO:0006880	intracellular sequestering of iron ion	2	0.0018
GO:0006749	glutathione metabolic process	3	0.00201
GO:0010150	leaf senescence	6	0.00231
GO:0042744	hydrogen peroxide catabolic process	2	0.00468
GO:0046677	response to antibiotic	7	0.00481
GO:0010200	response to chitin	4	0.00817
GO:0009651	response to salt stress	9	0.00861
GO:0017001	antibiotic catabolic process	2	0.01041
GO:0006575	cellular modified amino acid metabolic process	3	0.01397
GO:0009735	response to cytokinin	5	0.01497
GO:0006826	iron ion transport	2	0.01601
GO:0009751	response to salicylic acid	6	0.02073
GO:0045087	innate immune response	6	0.02138
GO:0009408	response to heat	4	0.02652
GO:0009920	cell plate formation involved in plant-type cell wall biogenesis	1	0.03201
GO:0006076	(1->3)-beta-D-glucan catabolic process	1	0.03829
GO:0009643	photosynthetic acclimation	1	0.03829
GO:0046482	para-aminobenzoic acid metabolic process	1	0.03829
GO:0016574	histone ubiquitination	1	0.03829
GO:0010039	response to iron ion	2	0.04304
GO:0010262	somatic embryogenesis	1	0.04454
GO:0043605	amide catabolic process	1	0.04454
GO:0031929	TOR signaling	1	0.04454
GO:0046902	regulation of mitochondrial membrane permeability	1	0.04454
GO:0071417	cellular response to organonitrogen compound	2	0.04931

Table S8. Numbers of shared plastic genes and genes likely to be involved in adaptation as well as those for cue-transfer, genetic adoption and pre-adaptive plasticity with α = 0.05 for differential expression tests compared to α = 0.1. Brackets denote the percentage of putatively adaptive genes with pre-adaptive plasticity, cue transfer and genetic adoption. Genetic adoption and pre-adaptive plasticity sets both increased by 1% at α = 0.1, and cue transfer decreased by 4% - i.e. minimal changes compered to α = 0.5. One pre-adaptive plasticity gene with no functional annotation was observed at α = 0.1.

Gene set	*n* at α = 0.05	*n* at α = 0.1
Coastal shared salt plasticity	957	1085
Mine shared salt plasticity	155	186
Coastal shared zinc plasticity	10933	10890
Mine shared zinc plasticity	143	190
evolved mine shared zinc plasticity	91	119
Constitutive evolutionary change	124	173
Cue transfer	28 (20%)	31 (16%)
Genetic adoption	30 (24%)	45 (25%)
Pre-adaptive plasticity	0 (0%)	1 (1%)

Table S9 Significantly enriched GO terms for cue transfer (number of terms = 20).

ID	Description	number of genes in GO	*p*-value
GO:0009635	response to herbicide	4	6.00E-10
GO:0072722	response to amitrole	3	2.30E-08
GO:0010272	response to silver ion	3	1.90E-07
GO:0043312	neutrophil degranulation	2	1.80E-05
GO:0006032	chitin catabolic process	2	1.80E-05
GO:0009627	systemic acquired resistance	3	3.20E-05
GO:0009615	response to virus	3	7.80E-05
GO:0009620	response to fungus	4	0.00019
GO:0007568	aging	3	0.00033
GO:0010150	leaf senescence	3	0.00038
GO:0051707	response to other organism	7	0.00111
GO:0006955	immune response	5	0.00186
GO:0006076	(1->3)-beta-D-glucan catabolic process	1	0.00592
GO:0046902	regulation of mitochondrial membrane permeability	1	0.00691
GO:0010262	somatic embryogenesis	1	0.00691
GO:0015867	ATP transport	1	0.01083
GO:0015866	ADP transport	1	0.01475
GO:1901679	nucleotide transmembrane transport	1	0.01767
GO:0009407	toxin catabolic process	1	0.02641
GO:0006749	glutathione metabolic process	1	0.03794

Table S10. Significantly enriched GO terms for genetic adoption (number of terms = 10).

ID	Term	Number of genes in GO	*p*-value
GO:0006970	response to osmotic stress	7	1.20E-05
GO:0010726	positive regulation of hydrogen peroxide metabolic process	1	0.011
GO:0009414	response to water deprivation	3	0.012
GO:0015700	arsenite transport	1	0.016
GO:0010200	response to chitin	2	0.017
GO:1902418	(+)-abscisic acid D-glucopyranosyl ester transmembrane transport	1	0.018
GO:0046355	mannan catabolic process	1	0.02
GO:0009409	response to cold	3	0.02
GO:0046685	response to arsenic-containing substance	1	0.026
GO:1901684	arsenate ion transmembrane transport	1	0.031

Table S11. Sample names and read counts (F+R) for salt experiment plants. RNA suffix indicates an individual plant sampled from living *S. uniflora* collections. Those with no suffix were more recently germinated for the experiment. Population codes are as follows: SA = Coast-W, BD = Coast-E, GR = Mine-W and PP = Mine-E.

Sample name	Plant name	Raw read counts F+R	Trimmed read counts F +R
SA02_C_7	SA 2	42335250	41749450
SA02_S_8	SA 2	40844158	40318092
SA04_C_15	SA 4	41093634	40623636
SA04_S_16	SA 4	41137778	40681912
SA07_C_11	SA 7	40270096	39773232
SA07_S_12	SA 7	41422260	40905500
GR-RNA-6_C_5	GR RNA 6	43722884	43224348
GR-RNA-6_S_6	GR RNA 6	42254266	41660636
GR-RNA-10_C_13	GR RNA 10	42256298	41771490
GR-RNA-10_S_14	GR RNA 10	41263170	40859672
GR-RNA-12_C_23	GR RNA 12	42829958	42299184
GR-RNA-12_S_24	GR RNA 12	44029256	43489784
BD03_C_17	BD 3	43535140	43087694
BD03_S_18	BD 3	41198658	40741972
BD05_C_9	BD 5	41026254	40539942
BD05_S_10	BD 5	42056414	41528166
BD07_C_19	BD 7	41615692	41156374
BD07_S_20	BD 7	43001934	42450886
PP1_C_3	PP 1	41515228	40961716
PP1_S_4	PP 1	39593352	39066246
PP12_C_21	PP 12	40989006	40379610
PP12_S_22	PP 12	41973704	41435506
PP-RNA-1_C_1	PP RNA 1	41920488	41382040
PP-RNA-1_S_2	PP RNA 1	40856702	40400804

Dataset S1 (SI_data_S1_ASP_annots.xlsx). Functional annotations for genes present in the shared coastal (ancestral) salt plasticity responses. Includes EggNOG orthologues, COG category, GO functions, Protein family names, and Kegg pathways. Produced using the *Silene uniflora* reference genome by Osborne *et al.* (10).

Dataset S2 (SI_data_S2_DSP_annots.xlsx). Functional annotations for genes present in the shared Mine (descendent) salt plasticity responses. Includes EggNOG orthologues, COG category, GO functions, Protein family names, and Kegg pathways. Produced using the *Silene uniflora* reference genome by Osborne *et al.* (10).

Dataset S3 (SI_data_S3_ECC_annots.xlsx). Functional annotations for genes present in the Evolved constitutive change gene set. Includes EggNOG orthologues, COG category, GO functions, Protein family names, and Kegg pathways. Produced using the *Silene uniflora* reference genome by Osborne *et al.* (10).

Dataset S4 (SI_data_S4_DZP_annots.xlsx). Functional annotations for genes present in the derived (mine) zinc plasticity gene set. Includes EggNOG orthologues, COG category, GO functions, Protein family names, and Kegg pathways. Produced using the *Silene uniflora* reference genome by Osborne *et al.* (10).

Dataset S5 (SI_data_S5_permutations.xlsx). All permutations of alternative patterns for putatively adaptive genes.

Dataset S6 (SI_data_S6_cue_transfer_annots.xlsx). Functional annotations for genes present in the cue transfer gene set. Includes EggNOG orthologues, COG category, GO functions, Protein family names, and Kegg pathways. Produced using the *Silene uniflora* reference genome by Osborne *et al.* (10).

Dataset S7 (SI_data_S7_gen_adopt_annots.xlsx). Functional annotations for genes present in the genetic adoption gene set. Includes EggNOG orthologues, COG category, GO functions, Protein family names, and Kegg pathways. Produced using the *Silene uniflora* reference genome by Osborne *et al.* (10).

Dataset S8 (SI_data_S8_salt_gene_count_matrix.csv). Raw gene count matrix generated in StringTie v2.2.0 (12) for salt experiment dataset.

Dataset S9 (SI_data_S9_zinc_gene_count_matrix.csv). Raw gene count matrix generated in StringTie v2.2.0 (12) for zinc experiment dataset.

Dataset S10 (SI_data_S10_experimental_setup.xlsx). Experimental set-up of salt and zinc samples used for input into DEseq2 datasets in R. Tab 1 is the salt experiment phenotype data, Tab 2 is the zinc experiment phenotype data and Tab 3 is the combined dataset for both experiments. The column Pop_treat shows the Population*treatment factor levels assigned for the first DEseq2 model structure. For combined experiment data, the salt control treatment was assigned the code C1 and zinc control was assigned the code C2.

SI References

1. D. P. Wood, *et al.*, Genetic assimilation of ancestral plasticity during parallel adaptation to zinc contamination in *Silene uniflora*. *Nat. Ecol. Evol.* **3**, 414-423 (2023).

2. A. S. T. Papadopulos, *et al.*, Rapid parallel adaptation to anthropogenic heavy metal pollution. *Mol. Biol. Evol.* **38**, 3724–3736 (2021).

3. A. Koźmińska, *et al.*, Comparative analysis of water deficit and salt tolerance mechanisms in *Silene*. *South Afr. J. Bot.* **117**, 193–206 (2018).

4. A. Koźmińska, *et al.*, Identification of salt and drought biochemical stress markers in several *Silene vulgaris* populations. *Sustainability* **11,** 800 (2019).

5. K. I. Köhl, NaCl homoeostasis as a factor for the survival of the evergreen halophyte *Armeria maritima* (Mill.) Willd. under salt stress in winter. *Plant Cell Environ.* **20**, 1253–1263 (1997).

6. L. Purmale, A. Jēkabsone, U. Andersone-Ozola, G. Ievinsh, Salinity tolerance, ion accumulation potential and osmotic adjustment *in vitro* and *in planta* of different *Armeria maritima* accessions from a dry coastal meadow. *Plants* **11**, 2570 (2022).

7. S. Andrews, FastQC: A quality control tool for high throughput sequence data. (2010). Deposited 2010.

8. P. Ewels, M. Magnusson, S. Lundin, M. Käller, MultiQC: summarize analysis results for multiple tools and samples in a single report. *Bioinformatics* **32**, 3047–3048 (2016).

9. A. M. Bolger, M. Lohse, B. Usadel, Trimmomatic: a flexible trimmer for illumina sequence data. *Bioinformatics* **30**, 2114–2120 (2014).

10. O. G. Osborne, *et al.*, Chromosome-scale genome assembly for *Silene uniflora*. *bioRxiv* (2024).

11. A. Dobin, *et al.*, STAR: ultrafast universal RNA-seq aligner. *Bioinformatics* **29**, 15–21 (2013).

12. S. Kovaka, *et al.*, Transcriptome assembly from long-read RNA-seq alignments with StringTie2. *Genome Biol.* **20**, 278 (2019).

13. M. I. Love, W. Huber, S. Anders, Moderated estimation of fold change and dispersion for RNA-seq data with DESeq2. *Genome Biol.* **15**, 550 (2014).

14. A. Alexa, J. Rahnenfuhrer, topGO: Enrichment analysis for gene ontology. (2023). Deposited 2023.

15. W. Walter, F. Sánchez-Cabo, M. Ricote, GOplot: an R package for visually combining expression data with functional analysis. *Bioinformatics* **31**, 2912–2914 (2015).
